# An integrated multi-variable optimization approach to tailor ankle-foot orthosis stiffness to end-user needs

**DOI:** 10.1186/s12984-026-02015-5

**Published:** 2026-05-14

**Authors:** Sejin Yi-Yoo, Emma A. Gille, Alejandro Dantart, Nikko Van Crey, Elliott J. Rouse, Edwin H. F. van Asseldonk, Cristina Bayón

**Affiliations:** 1 BioRobotics Group, Center for Automation and Robotics, Spanish National Research Council, CAR-CSIC-UPM, Madrid, Spain; 2 Universidad Politécnica de Madrid, Madrid, Spain; 3 Department of Biomechanical Engineering, University of Twente, Enschede, The Netherlands; 4 Departments of Robotics and Mechanical Engineering, University of Michigan, Michigan, USA

**Keywords:** Ankle-foot orthoses, Stiffness, Optimization, Gait

## Abstract

**Background:**

Ankle foot orthosis (AFOs) are devices commonly prescribed to assist or rehabilitate gait. A critical parameter influencing their effectiveness is the stiffness of the AFO. Although suppliers typically recommend stiffness levels based on general factors, such as body weight and activity level, these guidelines are insufficient to achieve optimal stiffness tailored to each individual. In this work, we introduce an integrated multi-variable optimization approach that simultaneously considers multiple aspects of gait. Unlike previous approaches that rely on a single performance metric (e.g., metabolic cost) or impose a predefined hierarchy among gait parameters, our method does not presume any predefined prioritization among the gait features selected. Furthermore, our approach allows the inclusion of users’ priorities, enabling a more personalized optimization of AFO stiffness.

**Methods:**

Ten children with cerebral palsy (CP) participated in an experimental protocol using the inGAIT-VSO, which is a passive variable-stiffness orthosis. The participants completed five separate 2-minute walking trials, each with a different stiffness configuration of the AFO. To determine the optimal stiffness for each participant within the tested configurations, we developed an optimization method that evaluates performance across five key gait domains: *kinematics*, *spatio-temporal*, *balance*, *user perception*, and *muscular control*. Furthermore, we investigated the integration of physiotherapists’ and users’ priorities into the optimization process.

**Results:**

The proposed optimization method identified the stiffness configuration for each child with CP that most closely aligned their gait to healthy patterns considering the five gait domains. The optimal stiffness varied not only across participants but also across gait domains within the same participant. These findings reveal the importance of having a multi-variable, user-tailored approach. Overall, the inclusion of physiotherapists’ and users’ priorities did not alter the optimal stiffness selection.

**Conclusion:**

Optimizing the preferred stiffness requires consideration of multiple variables. Our proposed method opens new possibilities for future research into the personalization and fine-tuning of AFO stiffness. In the future, this approach may benefit from expanded data collection efforts that enable a more efficient evaluation, for example by exploring the potential of deep learning models, which might support its integration into clinical practice.

**Supplementary Information:**

The online version contains supplementary material available at 10.1186/s12984-026-02015-5.

## Background

Ankle foot orthoses (AFOs) are devices commonly prescribed to assist or rehabilitate gait in people with neurological or motor disorders [1–5]. These devices support the ankle joint kinematics helping to correct irregular or abnormal walking patterns [6–8]. In addition, they enhance balance and postural control [5, 9], and promote more energy-efficient movement [4, 10] with positive effects on muscle activation [11, 12].

A key parameter influencing the performance of the user with an AFO is the AFO’s mechanical stiffness, which refers to the resistance of the AFO to deformation under load in both plantarflexion (PF) and dorsiflexion (DF). It determines how much flexibility, but also how much support the device provides during gait. For instance, in terms of joint kinematics, the AFO stiffness modulates the shape of the ankle motion profile [1], which in turn affects derived metrics such as peak ankle PF and DF angles [8]. Beyond joint-level effects, the AFO’s stiffness is also closely linked to broader biomechanical outcomes, including gait stability [5] and walking energy expenditure [4, 10, 13, 14].

Given its wide-ranging effects, optimizing AFO stiffness to match the individual user’s needs is essential for maximizing its effectiveness [1, 2, 4, 5]. However, determining the ideal stiffness is complex, as it depends on multiple factors such as the user’s physical capabilities [4, 5, 15], the specific task being performed [16, 17], or the terrain type [2, 18]. Additionally, current clinical guidelines lack standardized protocols for selecting optimal stiffness values tailored to individual users [3, 15, 19]. As a result, AFO prescriptions often rely on clinical judgment, guided by general manufacturer recommendations (e.g., based on a user’s weight) and patient evaluations conducted in controlled laboratory environments, which may not accurately reflect ambulation in the real world [3, 19–21]. Furthermore, existing approaches are hindered by practical challenges, including lengthy evaluation procedures and limited integration of user-specific variability [3, 19].

There have been some attempts to improve traditional methods for stiffness personalization. The most used approach is Human in the Loop Optimization (HILO), in which an objective function, commonly metabolic cost, is iteratively minimized [10, 13, 14, 22]. However, assessing metabolic cost typically demands extended measurement durations, and even when a metabolic benefit is achieved, users often do not consistently perceive this change [23, 24]. This discrepancy may stem from the fact that metabolic efficiency represents only one of the numerous factors that may improve gait performance, and for patients, it may not be the most relevant [24]. Consequently, focusing solely on optimizing a single physiological parameter may not lead to improvements in user experience or acceptance [25].

Recent studies have extended HILO to incorporate more than one objective [24, 26]. While these multi-objective HILO approaches represent a step forward, they remain constrained by practical challenges, including the need for long measurement sessions and limited scalability when the number of objectives increases, typically handling only two objectives effectively [24, 26].

Alternatively, decision scheme-based methods evaluate the performance across multiple objectives corresponding to different gait domains beyond effort, such as kinematics or spatio-temporal parameters [4, 15]. However, in these decision scheme methods, the primary objective function predominantly guides the decision-making process, while secondary objective functions serve as tie-breakers when necessary [4]. As a result, the hierarchical structure of the decision scheme imposes a fixed prioritization of the considered gait domains, regardless of individual user differences. This rigid prioritization may lead to inconsistent outcomes, as the relative importance of various gait domains can vary significantly across users [25].

As emphasized by Tankink et al. [27], the selection of cost functions (considering both the optimization objective and the variables involved) plays a crucial role in shaping the outcomes of assistive device tuning. This emphasizes that a “one-size-fits-all” approach may be suboptimal and challenges current optimization strategies, which often fail to capture the multifaceted nature of human gait and may not be universally applicable across users [10]. Supporting this, Pett et al. showed significant differences in preferred prosthesis stiffness across activities, with preferences varying by as much as 32$\%$ between users [28].

In this study, we propose a novel method for determining optimal AFO stiffness by simultaneously considering different gait domains without imposing a fixed hierarchy of priorities. We hypothesize that (1) the optimal stiffness selected by the multi-variable optimization will differ from the stiffness obtained when each gait domain or variable is considered independently; and (2) incorporating physiotherapists’ and patients’ priorities into the optimization will modify the selected optimal stiffness compared with the default (non-weighted) solution. Here we show the potential of this method for selecting the optimal AFO stiffness from a discrete set of tested stiffness levels in ten children with cerebral palsy (CP), and we assess how physiotherapist and user priorities influenced the final selection.

## Methods

### Variable stiffness orthosis

**Fig. 1:**
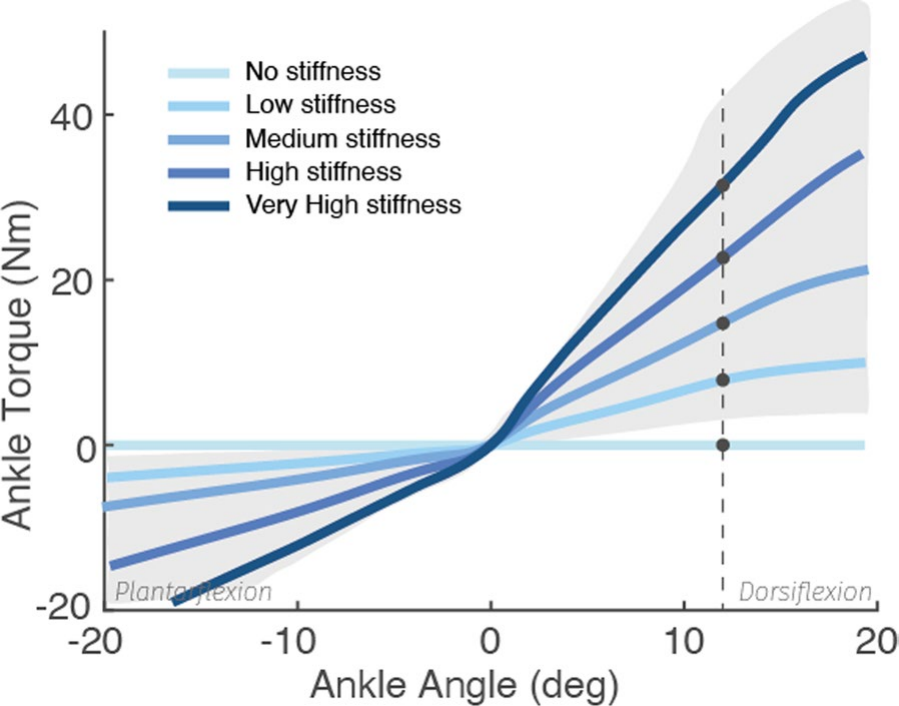
Graphical representation of the torque-angle curves for the five stiffness configurations of the inGAIT-VSO tested with a representative participant (CP01). Grey area indicates full range for stiffness selection

The inGAIT-VSO was the orthosis used to evaluate our stiffness selection method. This device is a pediatric version of the Variable Stiffness Orthosis (VSO) developed by Van Crey et al. [16], specifically designed for children with CP [8]. It incorporates a spring-based mechanism with a non-linear torque-angle relationship, which is active during both stance and swing phases. The overall stiffness of the torque-angle relationship can be manually adjusted to match the user’s particular needs. This stiffness influences both PF and DF movements from the neutral position (Fig. 1), helping to prevent drop-foot during swing and support body weight during stance, while also generating a restoring torque that contributes to ankle push-off.

For this study, we employed five different stiffness configurations, characterized by their effective stiffness at $12^\circ$ of DF within the AFO’s non-linear torque-angle profile (Fig. 1): (1) No stiffness – spring off, (2) Low stiffness – 0.017 $\frac{\textrm{Nm}}{\textrm{deg} \cdot \textrm{kg}}$, (3) Medium stiffness – 0.033 $\frac{\textrm{Nm}}{\textrm{deg} \cdot \textrm{kg}}$, (4) High stiffness – 0.050 $\frac{\textrm{Nm}}{\textrm{deg} \cdot \textrm{kg}}$, and (5) Very High stiffness – 0.067 $\frac{\textrm{Nm}}{\textrm{deg} \cdot \textrm{kg}}$. These five stiffness configurations were selected to span a clinically relevant range consistent with previous literature that tested a broad range of commercially available AFOs (0.6 – 3.4 $\frac{\textrm{Nm}}{\textrm{deg}}$ into DF) with multiple dynamometers [29], ensuring coverage from flexible to rigid AFO effects and minimizing the likelihood of large performance differences between untested intermediate stiffness levels [13, 30]. The value of $12^\circ$ DF was chosen based on prior work [8, 31]. For a detailed description of the inGAIT-VSO we refer to [8].

### Performance variables

To capture the multifaceted nature of gait, in our analysis we defined five gait domains: (1) kinematic features, (2) spatio-temporal metrics, (3) balance and stability, (4) user perception and physiological demand, and (5) muscle activation and control – hereafter referred to as *kinematics*, *spatio-temporal*, *balance*, *user perception*, and *muscular control*, respectively (see Table 1). Each gait domain included targeted performance variables (a total of 11) designed to evaluate both functional and physiological dimensions of movement. For the selection of the performance variables we followed outcomes from previous research [3, 21, 25, 32–38]. Our rules for inclusion aimed to avoid redundancy, duplication and overlap both within and across gait domains. We prioritized variables sensitive to gait impairments, with particular emphasis on the ankle joint, and measurable using wearable sensors or standard laboratory tools.

Each performance variable was computed at one of the following computational intervals: every gait cycle, walking segment or walking trial (see Table 1). Each walking trial consisted of walking back and forth in a 20-meter straight path for 2 min, and each walking segment referred to the straight portion of the path, excluding the turns.

**Table 1:** Summary of performance variables and their corresponding domain, gait aspects, typical developing (TD) values, acceptable bounds (as obtained from the literature), and the computational interval, with “W.S.” referring to walking segment and “W.T.” to walking trial

Domain	Gait aspect	Performance variables	TD values	Bounds	Interval
Kinematic features	Amplitude	Strike ankle dorsiflexion (deg)	−2.15	[−5.9, 1.6]	Gait cycle
		Peak ankle dorsiflexion (deg)	12.5	[9.5, 15.5]	Gait cycle
Spatio-temporal metrics	Pace	Walking speed (m/s)	$\ge 1.2$	[1, 1.4]	Gait cycle
	Rhythm	Stance duration (%)	57.97	[56, 59.9]	Gait cycle
	Variability	Stance duration variability (%)	$\le 1.93$	[0, 1.93]	W.S
	Coordination	Walk ratio (cm$\cdot$min/step)	0.45	[0.38, 0.53]	Gait cycle
Balance and stability	Body compensation	Trunk lateral motion (m/s^2^)	$\le 1.47$	[1.08, 1.86]	Gait cycle
	Asymmetry	Step time asymmetry (ms)	$\le 32$	[25.6, 38.4]	W.S
User perception and physiological demand	Effort	Physiological cost index (beats/m)	$\le 0.39$	[0.3, 0.48]	W.S
		Perceived effort (Borg’s scale)	$\le 12$	[6, 12]	W.T
Muscle activation and control	Smoothness	Co-contraction index (–)	26.5	[24, 29]	Gait cycle

In the *kinematics* domain, we assessed the amplitude of ankle movement by extracting the ankle DF angle at two representative instants of each gait cycle [3]: at heel strike and at its peak value during the stance phase. These variables reflect critical information related to impaired initial contact, capacity for propulsion and forward progression.

For the *spatio-temporal* domain we evaluated pace, rhythm, coordination, and variability. Pace was quantified by walking speed, as it reflects overall gait performance and is sensitive to motor impairments [21]. Rhythm was represented by the stance phase duration (as a percentage of the gait cycle), a common indicator of temporal gait organization [3, 21]. Gait variability was captured through stance duration variability, which tends to be higher in children with CP compared to TD peers [21, 32]. To assess coordination, we used the walk ratio (step length divided by cadence), which is considered independent of walking speed and has been shown to reflect motor control and coordination, especially in children with neurological disorders [21, 33].

In the *balance* domain, we analyzed body compensation and asymmetry. Trunk lateral motion, computed as the root mean square of lateral acceleration, served as a proxy for compensatory movements to keep balance [21, 34]. Step time asymmetry (STA) was used to quantify gait asymmetry, a marker of gait efficiency particularly in unilaterally affected patients [21, 35]. It was calculated as:

1 $$\begin{aligned} STA = \dfrac{\sum _{k=2}^{K} |ST_k - ST_{k-1}|}{2K} \end{aligned}$$

where $ST_k$ denotes the step time of the $k-th$ step (milliseconds), and $K$ represents the total number of steps within the corresponding 20-meter walking segment.

The domain *user perception* included measures of effort. The Physiological Cost Index (PCI) [36] was calculated for each 20-meter walking segment to estimate energy expenditure:

2 $$\begin{aligned} PCI = \dfrac{{HR}_{walk} - {HR}_{rest}}{\dot{x}} \end{aligned}$$

where ${HR}_{{walk}}$ and ${HR}_{{rest}}$ are the heart rate measurements during walking and at rest, respectively, and $\dot{x}$ represents the walking speed. Moreover, the Borg’s scale [37] was used to report perceived exertion.

Lastly, for the *muscular control*, we assessed smoothness using the co-contraction index (CCI) during stance phase, which reflects the degree of simultaneous activation of antagonist muscles and is indicative of motor control strategies [38]:

3 $$\begin{aligned} CCI = \sum _{i=1}^{101} \frac{{EMG}_L(i)}{{EMG}_M(i)} ({EMG}_L(i) + {EMG}_M(i)) \end{aligned}$$

where i represents the individual time points of stance phase (resampled into 101 data points), and ${EMG}_L(i)$ and ${EMG}_M(i)$ are the normalized activations of the less and more active muscles at point $i$, respectively. The antagonist muscles, tibialis anterior (TA) and gastrocnemius medialis (GM) were used for this computation.

### Optimal stiffness selection

Our optimization method involved three main steps. First, we extracted the individual performance variables from the data, as described in Section “Performance variables”. Second, we computed their alignment with normative healthy values in different domains and aggregated them into a single global metric, which served as the cost function. Finally, we identified the optimal stiffness configuration as the one that resulted in the best performance (i.e., the lowest cost function value) among the discrete set of tested stiffness configurations.

In our testing scenario with children with CP, we used typically developing (TD) values as a reference (see TD values and acceptable bounds in Table 1). This decision was motivated by the assumption that optimizing toward TD patterns across multiple gait domains can promote a more efficient, stable, and functionally effective movement. The acceptable TD bounds (Table 1) were obtained from literature, primarily considering data from TD children’s gait and, in their absence, from healthy adults. Specifically, the following references were used to extract TD values for each variable: strike ankle DF, peak ankle DF, walking speed, stance duration, stance duration variability, and walk ratio, all from [39]; trunk lateral motion from [34]; STA from [35]; PCI from [8]; perceived effort from [37]; and CCI from [40].

To determine the averaged individual contribution of each variable to the overall closeness to the TD values, the root mean squared error (RMSE) was calculated for each variable as follows:

4 $$\begin{aligned} {RMSE}^{(v)}(s) = \sqrt{\frac{1}{N} \sum _{n=1}^{N} \left( f\left( X_{TD}^{(v)}, X_{CP}^{(n,v)}(s)\right) \right) ^2} \end{aligned}$$

where *v* is the individual performance variable (from Table 1), *s* is the stiffness configuration used in the trial, *N* is the total number of gait cycles within the trial, $X_{TD}^{(v)}$ is the TD value for the said variable, $X_{CP}^{(n,v)}(s)$ is the computed value of the variable for each of the gait cycles, and *f* is an error function that was defined based on the characteristics of the performance variables in three ways:

- For the performance variables strike ankle DF, peak ankle DF, stance duration, walk ratio, and CCI, deviations in either direction of the TD value contributed to the error:
  5 $$\begin{aligned} f(X_{TD}, X_{CP}) = X_{TD} - X_{CP} \end{aligned}$$
- For walking speed, exceeding the TD value was not penalized because higher speeds were considered beneficial. Consequently, only values lower than the TD value were penalized:
  6 $$\begin{aligned} f(X_{TD}, X_{CP}) = {\left\{ \begin{array}{ll} X_{TD} - X_{CP} & \text {if } X_{CP} < X_{TD} \\ 0 & \text {if } X_{CP} \ge X_{TD} \end{array}\right. } \end{aligned}$$
- Lastly, for variables where a reduction was generally beneficial, such as stance duration variability, trunk lateral motion, STA, PCI, and perceived effort, only values exceeding the TD value were penalized:
  7 $$\begin{aligned} f(X_{TD}, X_{CP}) = {\left\{ \begin{array}{ll} 0 & \text {if } X_{CP} < X_{TD} \\ X_{TD} - X_{CP} & \text {if } X_{CP} \ge X_{TD} \end{array}\right. } \end{aligned}$$

In order to ensure the integration of the RMSE values of all the variables into a single global metric, each averaged variable was first divided by a normalization term as so:

8 $$\begin{aligned} \overline{\textit{RMSE}}^{(v)}(s) = \frac{{RMSE}^{(v)}(s)}{b_{\text {high}} - b_{\text {low}}} \end{aligned}$$

where $b_{high}$ and $b_{low}$ are the high and low bounds observed in natural gait of TD children (Table 1). This operation rendered error values unitless and standardized, preventing biases due to the variable’s magnitude and inherent variability.

The normalized errors ($\overline{\textit{RMSE}}$) of all variables were summed into a single global metric that represents the total error for each stiffness configuration. The configuration with the lowest total error was selected as the optimal stiffness:

9 $$\begin{aligned} s^* = \arg \min _{s \in \mathcal {S}} \sum _{v=1}^{11} \overline{\textit{RMSE}}^{(v)}(s) \end{aligned}$$

where $s^*$ is the optimal stiffness and $\mathcal {S}$ is the subset of tested stiffness configurations.

### Physiotherapist- and User-weighted stiffness selection

To incorporate both physiotherapist and user priorities regarding the different gait domains, each variable’s error value was weighted based on priority ratings collected through a custom-designed questionnaire (see English translation in Appendix A). The questionnaire comprised two components: (1) a Likert scale where responders rated the importance of each gait domain from 1 (unimportant) to 5 (very important), and (2) a ranking task in which they selected their top three prioritized domains in order of importance. Identically structured questionnaires were administered to individuals with CP and physiotherapists, with the only distinction being language adaptations tailored to each group.

The questionnaire responses were converted into numerical weights to reflect the relative importance of each gait domain. For the Likert scale we used weights ranged from 0.2 to 1 in increments of 0.2, depending on the importance given. Additionally, for the ranking task, bonus weights of 1.0, 0.8, and 0.6 were assigned to the first, second, and third most prioritized domains, respectively, to ensure that the top three selected priorities had a proportionally greater influence on the final weighting. The total weight for each domain (*w*) was normalized by the number of variables within that domain. This normalization prevented disproportionate weighting of domains with more variables:

10 $$\begin{aligned} w^{(v)} = \frac{w_{likert}+w_{ranking}}{V_{domain }}+\alpha \end{aligned}$$

where $w_{likert}$ is the Likert scale weight, $w_{ranking}$ is the ranking weight, $V_{domain}$ is the number of variables inside a specific domain, and $\alpha$ is an influence modulation term added to control the impact of the weight on the final error calculation. As default value, $\alpha = 1$.

Finally, the weights were incorporated into equation (9) as:

11 $$\begin{aligned} s^*_{weighted} = \arg \min _{s \in \mathcal {S}} \sum _{v=1}^{11} w^{(v)} \cdot \overline{\textit{RMSE}}^{(v)}(s) \end{aligned}$$

### Experimental protocol

#### Participants

Ten children with CP took part in a pilot-study to test the optimization model (weight 34.35 ± 14.61 kg, height 1.39 ± 0.13 m, age 10.40 ± 3.01 years old) (Table 2). Participants met the following inclusion criteria: aged 5–17 years, predominantly spastic uni- or bilateral CP, Gross Motor Function Classification System (GMFCS) [41] levels I–III, sufficient cognitive ability, and the capacity to complete the walking protocol. Flexible equinus deformities or drop-foot were preferred, with Ashworth scale scores ranging from 1 to 3. Exclusion criteria included leg surgery in the previous 6 months or botulinum toxin A injections in the previous 3 months, significant musculoskeletal deformities, unhealed skin lesions, gastrocnemius shortening $>\!10^\circ$, or visual impairments or behavioral problems that could hinder protocol performance. All participants were prescribed an AFO by a medical professional. However, CP02, CP08 and CP09 ceased daily use of their AFOs for various reasons, including personal choice and outgrowth. The remaining participants continued to wear their prescribed AFOs daily.

The Local Ethics Committee at Hospital Infantil Universitario Niño Jesús (HNJ) gave approval (R-0064/23) and ensured the study was conducted in alignment with the Declaration of Helsinki. Participants and their families were informed accordingly, and both participants’ assent and parental consent were obtained prior to participation.

#### Experimental procedure

**Fig. 2:**
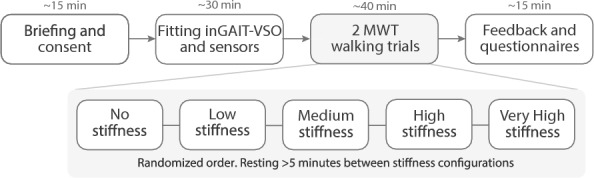
Experimental protocol overview

Each participant completed a single $\approx$2-hour session. The inGAIT-VSO was fitted bilaterally or unilaterally according to clinical presentation, diplegia, or hemiparesis, respectively. After a brief familiarization period ($\approx$1–3 min), during which participants walked with the inGAIT-VSO and, if needed, had the equipment adjusted, they performed five trials of 2-minute walking test (2MWT, [42]) along a 20-meter corridor. Each trial used a different stiffness configuration as described in Sect. “Variable stiffness orthosis”. Trial order was randomized, with rest periods allowed between trials. This protocol, combined with the initial familiarization, was designed to ensure that participants were sufficiently accustomed to the device before data collection began, and to minimize any systematic bias related to trial sequence. After finishing the walking trials, participants and their physiotherapists completed the priority questionnaires (Sect. “Physiotherapist- and User-weighted stiffness selection”). A flowchart of the protocol is shown in Fig. 2.

**Table 2:** Participants’ characteristics

Participant ID	Age (years)	Weight (kg)	Height (m)	Shoe size (EU)	Clinical manifestation	Pattern	GMFCS	Sex	Daily-use orthosis
CP01	9	38	1.39	36	Hemiparesis (left)	Equinus	I	Male	Articulated AFO
CP02	10	24	1.34	33	Spastic diplegia	Crouch	III	Female	-
CP03	14	74	1.69	41	Hemiparesis (right)	Drop foot	I	Female	Rigid AFO
CP04	5	26	1.20	31	Hemiparesis (right)	Drop foot	I	Male	Articulated AFO
CP05	9	23	1.30	32	Spastic diplegia	Equinus	II	Male	Rigid AFO
CP06	8	29	1.31	32	Hemiparesis (right)	Drop foot	I	Female	Articulated AFO
CP07	12	27.5	1.40	34	Hemiparesis (left)	Drop foot	I	Male	Rigid AFO
CP08	12	26	1.40	36	Hemiparesis (right)	Equinus	II	Male	-
CP09	9	32	1.36	36	Hemiparesis (right)	Equinus	I	Male	-
CP10	16	44	1.54	39	Spastic diplegia	Crouch	II	Male	Rigid insole

#### Data acquisition

During the 2MWTs, a multi-sensor setup was used to capture the performance variables listed in Table 1. Ankle angles in the sagittal plane were recorded at 100 Hz using magnetic encoders (AS5048b, AMS-OSRAM AG, Premstaetten, Austria) integrated into the inGAIT-VSO. Heel strike and toe-off events were detected using force-sensitive resistors (FSRs, FlexiForce A502, Tekscan Inc., MA, USA) embedded in the insoles, also sampled at 100 Hz [8]. Foot motion was tracked using two GaitUp Physilog sensors (Lausanne, Switzerland) attached to the shoes operating at 128 Hz, which specially served to calculate *spatio-temporal* performance variables. Surface electromyography (EMG) signals were collected bilaterally from the TA, GM, soleus (SOL), and gastrocnemius lateralis (GL) muscles using Trigno sensors (Delsys, Natick, MA, USA) at a sampling rate of 1926 Hz. Trunk motion was monitored via an accelerometer sensor (Fitbit Versa 3, San Francisco, USA) placed on the chest, recording at 100 Hz. Additionally, heart rate was measured at 1 Hz using a smartwatch (Fitbit Versa 3, San Francisco, USA). After each trial, participants rated their perceived exertion using the Borg scale [37].

#### Data processing

Data from the inGAIT-VSO, EMG sensors, IMU sensor, and smartwatch was processed using MATLAB 2021b (MathWorks, Natick, MA, USA). Data from the GaitUp sensors was processed using GaitUp Lab (Physilog, Lausanne, Switzerland). EMG data was preprocessed to remove noise and artifacts. This process consisted of bandpass filtering with cutoff frequencies at 30 and 300 Hz, full-wave rectification, and low-pass filtering with a cutoff frequency at 3 Hz. The resulting linear envelopes were normalized to the maximum activation value observed during the No stiffness walking trial.

After preprocessing, all data was resampled into a common frequency, synchronized and segmented into gait cycles based on heel strike events detected with the FSRs. Each gait cycle was linearly interpolated to 300 data points. Variables were calculated as described in Section “Performance variables” with a computation interval specified in Table 1.

## Results

The experimental protocol was conducted with no adverse events for the participants. As CP03 exceeded the inGAIT-VSO’s weight limit of 60 kg, this participant did not perform the High and Very High stiffness configurations. Additionally, due to issues with data collection, for CP06 we had to discard the data of No, High and Very High stiffness configurations.

An average of 116±17 gait cycles were recorded per participant and stiffness configuration during the 2MWTs. Performance variables within the *kinematic features* domain were calculated unilaterally for the affected side in participants with hemiparesis and bilaterally for participants with diplegia.

### Optimal stiffness selection

**Fig. 3:**
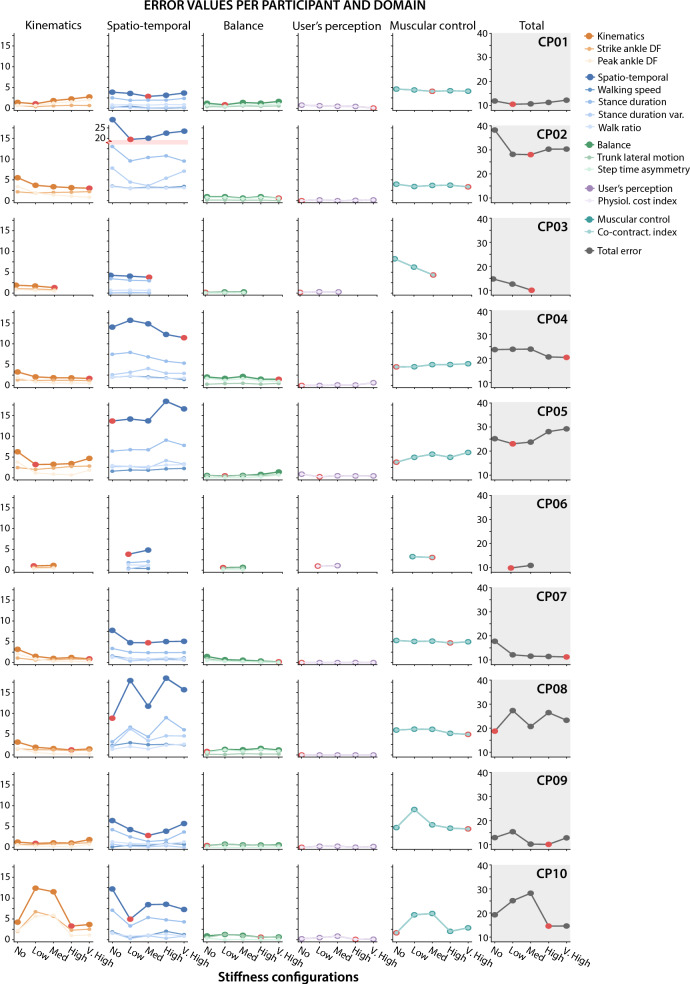
Error values for each participant with CP relative to TD children. Each row of subplots corresponds to a participant, and each column represents a gait domain. The last column (with a grey background) shows the total error across all domains. For performance variables measured bilaterally values are combined into a single line. Red dots indicate minimal error within each domain and the optimal stiffness ($s^*$) for each participant in the total error subplot

The proposed optimal stiffness selection process resulted in an individualized stiffness configuration for each participant, minimizing divergence from TD children’s gait across gait domains (Fig. 3). The optimal stiffness configurations ($s^*$) identified were: No stiffness for CP08, Low stiffness for CP01, CP05 and CP06, Medium stiffness for CP02 and CP03, High stiffness for CP09 and CP10, and Very High stiffness for CP04 and CP07. In all cases but for CP08, the stiffness configuration immediately adjacent to the optimal one yielded error values that were very close to those obtained with the optimal configuration (Fig. 3). However, as the stiffness deviated further from the optimal configuration, the associated errors increased, indicating the sensitivity of gait performance to stiffness selection.

The total error magnitude varied across participants, with CP02 exhibiting the largest deviation from TD gait and CP06 the lowest (Fig. 3). This trend aligned with the participants’ GMFCS level: participants classified as GMFCS II and III (i.e., CP02, CP05, CP08 and CP10) exhibited greater deviations from TD gait, while those GMFCS I typically showed smaller differences.

The domain-based errors varied within and across participants, indicating that the deviations were not uniform across all gait aspects and they were also participant-dependent (Fig. 3). Overall, the domains with larger errors were the *kinematics*, *spatio-temporal* and *muscular control*.

The effect of different stiffness configurations on performance errors varied both across participants within the same domain and across domains within the same participant. For example, while both CP04 and CP05 exhibited high errors in the *spatio-temporal* domain, increasing stiffness led to a reduction in error for CP04 but an increase for CP05 (Fig. 3). Similarly, in the case of CP10, the Low stiffness configuration minimized error in the *spatio-temporal* domain but simultaneously resulted in higher errors in the *kinematics* and *muscular control* domains.

### Physiotherapist- and user- weighted stiffness selection

**Fig. 4:**
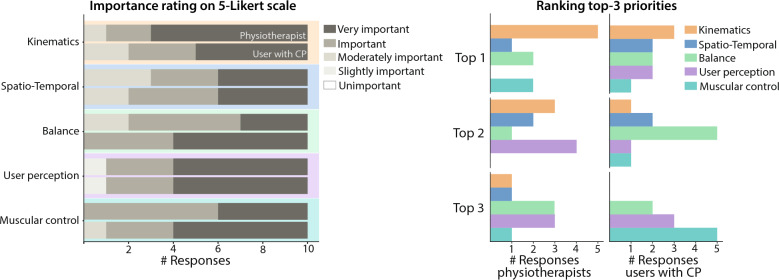
Physiotherapists’ and users’ domain-based priorities responses. Left: Responses to the Likert scale question indicating absolute importance given to each domain. Right: Responses to the Ranking question indicating relative importance given to each gait domain denoted by first, second and third most important

**Fig. 5:**
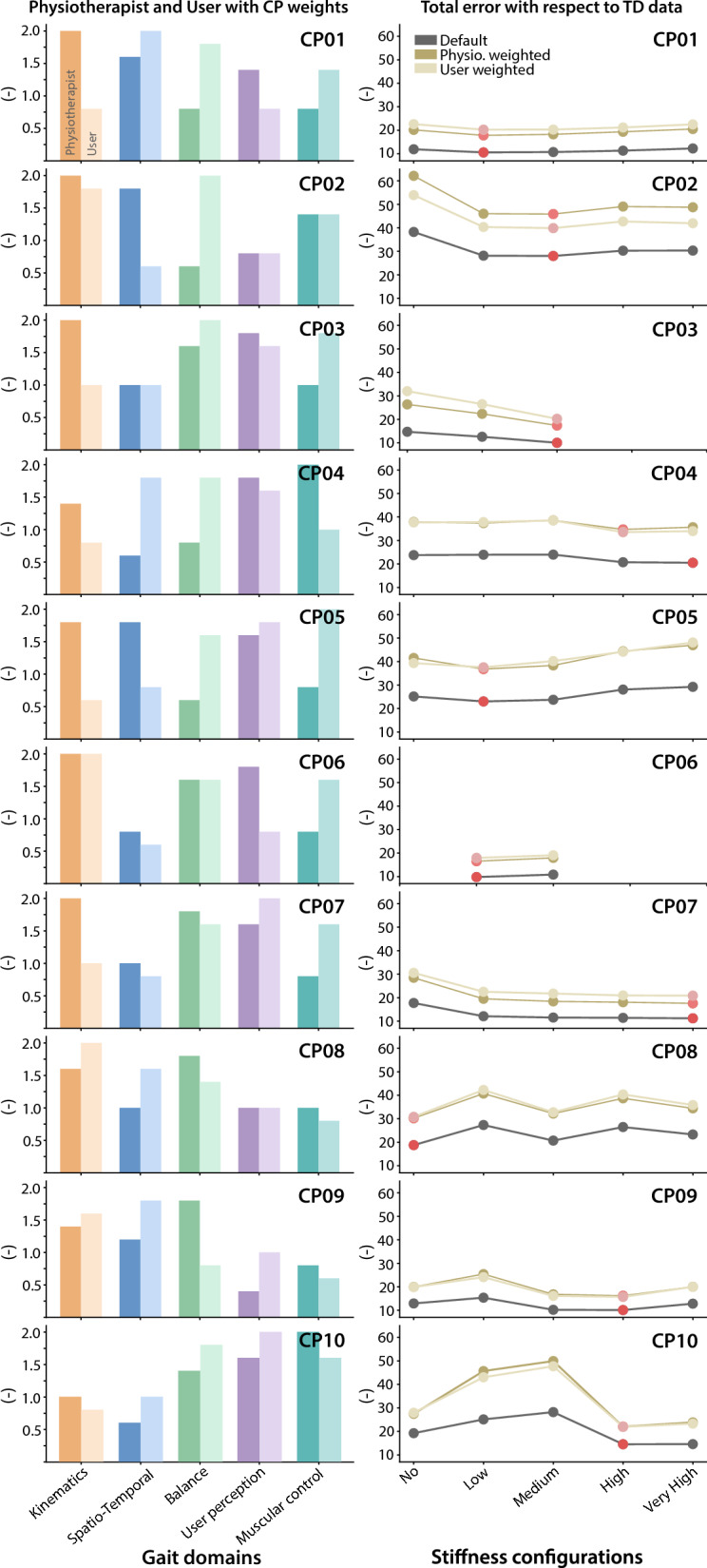
Left: physiotherapists’ and users’ weights given to each domain. Right: default (grey), physiotherapist-weighted (dark yellow) and user-weighted (light yellow) total error per stiffness configuration. Red dots represent optimal stiffness configurations for each participant

Questionnaires given to assess physiotherapist and user priorities revealed differences between these two populations (Figs. 4 and 5-left). All users with CP ranked the *balance* domain among their top three priorities, and 8 out of 10 also included *muscular control*. In contrast, only five physiotherapists prioritized *balance*, and just two included the *muscular control*. Conversely, *kinematics* was ranked by 9 out of 10 physiotherapists while only 3 users considered it a priority (Fig. 4-right). These discrepancies were particularly evident in cases such as CP03, CP05, and CP07 (*kinematics* vs *muscular control*), and CP04 and CP05 (*kinematics* vs *balance*) (Fig. 5-left).

Both physiotherapists and users priorities were incorporated into the optimal stiffness selection process by upweighting the error values of the domains of interest (Eq. 11). With the exception of CP04, the weighted optimal stiffnesses ($s^*_{weighted}$) matched the default optimal stiffness ($s^*$) across all participants (Fig. 5-right). For CP04, both the physiotherapist and user $s^*_{weighted}$ shifted the $s^*$ from Very High to High stiffness. However, in this case, the difference in total error between $s^*$ and $s^*_{weighted}$ was very low.

## Discussion

This study aimed to propose a method for identifying the optimal AFO stiffness that captures multiple gait domains and is applicable to participants with diverse characteristics. Our method reported an individualized optimal stiffness value for each participant with CP by minimizing deviations from TD gait, without assuming any predefined priority among gait domains. Additionally, the study explored how incorporating the priorities of physiotherapists and users into the optimization process influenced the final stiffness selection.

### Optimal stiffness selection

By applying our multi-variable optimization method, we identified a personalized stiffness configuration (among the discrete levels tested) for each participant with CP that minimized total error relative to TD gait. Our approach moves beyond traditional methods such as visual gait assessment and patient-reported perceptions and feelings, and introduces a more structured and quantitative framework for stiffness selection. Nonetheless, while this represents a promising step toward individualized tuning, assessing the true success of the optimization remains challenging due to the lack of a clearly defined ground truth [3, 20] and the absence of direct validation through real world performance measures.

Interestingly, the stiffness configuration that minimized error varied across gait domains within the same participant, highlighting the importance of considering multiple gait domains to capture a comprehensive view of gait performance. It is noticeable that for some cases (i.e., CP02 and CP09), the final optimal stiffness configuration did not correspond to any of the domain-specific optima (see Fig. 3). These results remark the limitations of methods that rely on a cost function with a single variable (e.g., HILO [10, 13, 14, 22]), because as previously noted by Tankink et al. [27], optimization outcomes can differ significantly depending on the domain selected. Moreover, unlike decision-scheme-based methods that apply a fixed prioritization of domains [4, 15], our approach adapts to individual gait patterns by assigning importance to several domains simultaneously, potentially resulting in a more personalized and clinically meaningful outcome. However, further validation is needed to confirm its clinical effectiveness.

Across participants, the *kinematics*, *spatio-temporal* and *muscular control* domains consistently provided larger error values, whereas the *balance* and *user perception* showed consistently lower errors. Several factors might explain this pattern. One possibility may be the limited sample size of our study, which may have reduced the likelihood of capturing participants whose dominant impairments lie outside the three primary domains. Additionally, the lower errors in the *balance* domain may be partially attributed to sensor placement. The sensor used to measure trunk lateral motion was positioned on the chest. However, a higher placement on the upper sternum would likely have captured lateral sway more accurately, as medio-lateral displacement increases with height along the trunk. Regarding the *user perception* domain, the self-selected walking pace during the 2MWTs might have allowed participants to modulate their pace to avoid excessive fatigue. Despite these considerations, a trend was observed between the magnitude of the computed errors and the participants’ GMFCS levels, with higher errors generally associated with higher GMFCS levels. An exception was participant CP04 (GMFCS I), whose relatively large errors may be attributed to their young age (5 years old).

It is important to note that the computed error values are not directly comparable across participants due to differences in the number of performance variables included in the analysis: participants CP02, CP05 and CP10 wore the inGAIT-VSO bilaterally, resulting in bilateral computation of the *kinematic* variables, and consequently, it increased the total number of variables considered for these individuals. Additionally, the computed error values are also not comparable across domains, as the number of performance variables varied depending on the domain. This variation occurred because some domains required more variables to be fully represented, while others could be adequately characterized with fewer. Importantly, all variables were carefully selected to avoid redundancy, ensuring that each contributed with unique and meaningful information to the optimization process.

### Physiotherapist- and user- weighted stiffness selection

As previously reported in [3], our findings also revealed a divergence between physiotherapists’ and users’ priorities (Fig. 4). This divergence likely arises from the different perspectives and goals of each group: while physiotherapists may prioritize kinematics outcomes, users often place greater value on factors such as comfort, safety and independence.

Despite the differences in priorities between physiotherapists and users, and contrary to our expectations, incorporating these priorities into the optimization process resulted in the same weighted optimal stiffness ($s^*_{weighted}$) for both groups. For the majority of participants, $s^*_{weighted}$ matched the default optimal stiffness ($s^*$) derived from the unweighted data, indicating that the inclusion of priorities had limited impact on the final selection. This differs from previous studies, such as Shepherd et al. [43], which reported that prosthetics’ preferred stiffness values were on average 26% higher than those preferred by amputee users. However, in our case, we did not directly assess stiffness preferences (i.e., user-driven preference tuning), but instead optimized stiffness by incorporating users’ functional weighted priorities into our performance-driven optimization framework. Our findings suggest that the prioritized domains were either already influential in the original optimization or showed minimal performance deficits (i.e., low error relative to TD reference values), which reduced the impact of increasing their weighting on the final optimization outcome. The only exception was participant CP04, for whom the optimal stiffness shifted from Very High to High. However, the difference in total error between these two configurations was minimal. Importantly, the priority-weighting process effectively emphasized the selected domains without disregarding others or compromising the overall optimization. For instance, in CP04, the High stiffness configuration (weighted optimal stiffness) reported lower errors than the Very High configuration in the physiotherapist-prioritized domains (i.e., *user perception* and *muscular control*, Fig. 3).

Although, in this case, incorporating the priorities of physiotherapists and users did not produce a clear effect on the final optimal stiffness selection, their input remains essential and should be carefully considered in the decision-making process [16, 17, 44]. One way to enhance the influence of these priorities within the optimization is by adjusting the influence term $\alpha$ (Eq. 10). Lower values of $\alpha$ increase the relative effect of the prioritized domains, resulting in a more dominant role in the optimization outcome (see an example in Appendix B). However, adjustments to this parameter should be made with caution. Excessive reduction of $\alpha$ may diminish the contribution of other relevant gait domains, potentially leading to an incomplete or biased stiffness selection that overlooks important aspects of the participant’s overall gait profile.

### Study limitations and future work

One of the main limitations of this study is the small sample size (ten participants with CP), which restricts the generalizability of the findings. Nevertheless, the results still offer valuable insight. Despite the limited cohort, a high degree of inter-individual variability was observed in how different gait domains responded to stiffness changes, reinforcing the need for a multi-variable, user-tailored optimization approach.

The Borg scale, used to estimate perceived effort, was excluded from the optimization due to bias in registering this variable. Because of the participants’ young age, the correct understanding and objective reporting of this metric could not be guaranteed. Some participants repeated the same value across all stiffness configurations, while others appeared to adjust their ratings based on the (randomized) trial order, suggesting a comparative rather than absolute perception of effort.

In this study we focused on five gait domains chosen based on previous literature [3, 21, 25, 32–38], and on acute responses to different stiffness configurations. Data from other potentially informative sources such as kinetics or kinematics of additional joints (e.g., hip or knee) were not included due to the lack of appropriate measuring and portable equipment. Although the inclusion of additional sources may offer further insight into gait performance and optimization, it is worth noting that previous studies using similar AFOs have demonstrated minimal changes in the kinematics of the knee or hip, with the AFO effects primarily localized at the ankle level [17]. Nevertheless, future work should consider both longer acclimation periods to assess adaptation to the stiffness configurations and the integration of additional joint data to enhance the comprehensiveness of the analysis.

An additional concern is whether a 2MWT is sufficiently long for all performance variables to reach stabilized values (e.g., heart rate–based measures such as PCI). The 2MWT is a validated and reliable test in children with neuromuscular disorders [42], and its use in our protocol reflects a balance between obtaining meaningful gait data and ensuring feasibility when multiple stiffness conditions must be tested within a single session. Longer walking tests such as the 6MWT could impose larger physical burden for the participants, being unfeasible for this population. Future work could explore whether longer-durations or test-retests influence the optimization outcome.

Lastly, the proposed method requires extensive data collection for each individual, as performance variables must be computed across all tested stiffness configurations (five in this study). This process can be time-consuming and resource-intensive, which may limit its practicality in clinical settings. To address this, future work could focus on expanding the dataset with more participants, enabling the development of AI-driven approaches such as deep learning models that could reduce the number of required stiffness trials while still accurately identifying the optimal configuration. This would enhance the feasibility and efficiency of the method for real-world applications.

## Conclusion

This study presented an integrated multi-variable approach for ankle-foot orthosis stiffness optimization. Our results show that each gait domain contributed uniquely to the optimal stiffness selection and that these contributions varied across participants. We highlight the importance of having a multi-variable user tailored stiffness optimization, as focusing on a single domain or generalizing the approach may provide an incomplete or even misleading assessment of individual gait performance. Future work may focus on the extension of our proposal using deep learning methods to simplify the data collection process, facilitating the integration of this approach into clinical practice.

## Supplementary Information

Below is the link to the electronic supplementary material.

Supplementary Material 1

## Figures and Tables

**Fig. 6:**
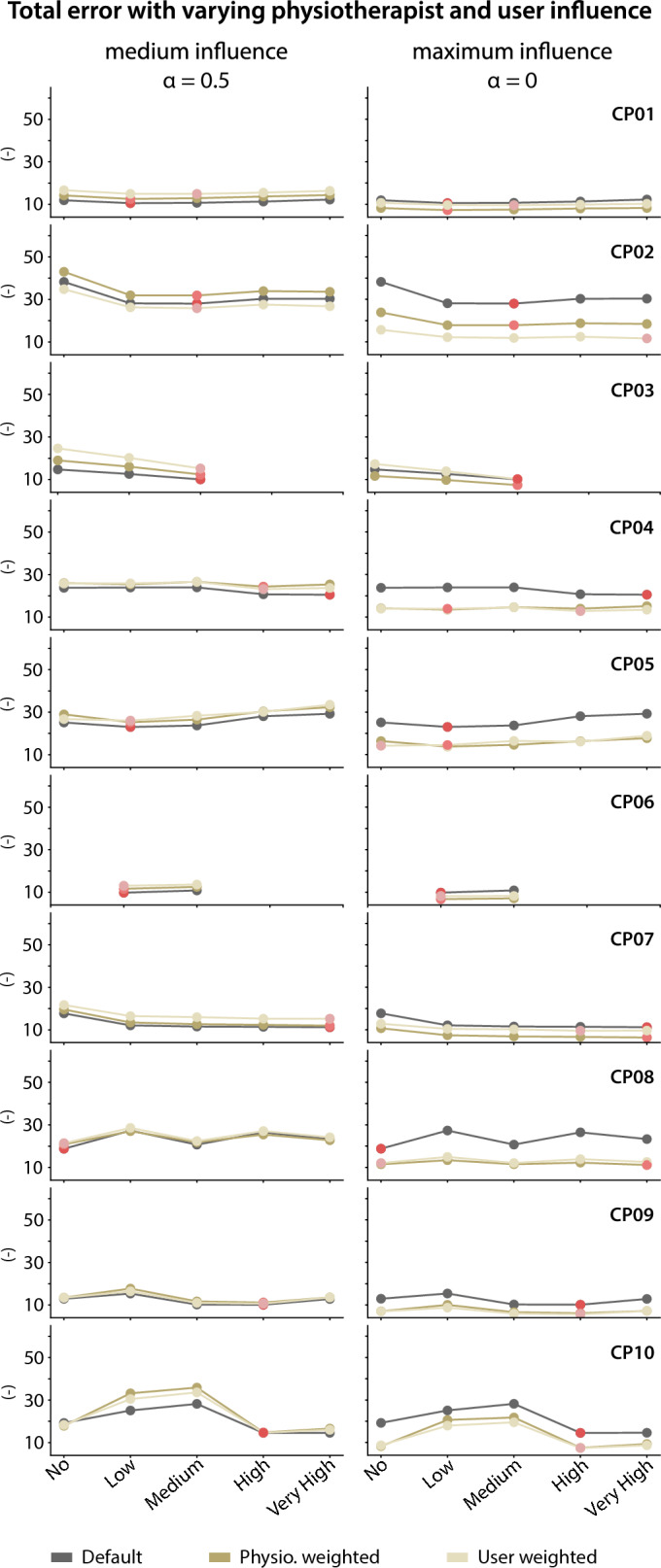
Default (grey), physiotherapist-weighted (dark yellow) and user-weighted (light yellow) total error per stiffness configuration for $\alpha =0.5$ (left) and $\alpha =0$ (right). Red dots represent optimal stiffness configurations for each participant

## Data Availability

Important data is provided within the manuscript or supplementary information files. The rest of anonymized datasets generated and/or analyzed during the current study are available from the corresponding author on reasonable request.

## Appendix A

A translation of the questionnaire given to the participants with CP is provided below. The questionnaire included illustrative graphics to facilitate the understanding of the questions. An equivalent questionnaire was provided to the physiotherapists, with few adaptations for more specific and technical language.

Table 3 presents a summary of the individual responses of each participant with CP and their physiotherapist to the provided questionnaire.

**Table 3:** Physiotherapist and user priorities reported through Likert ratings and ranking tasks across gait domains

Participant ID	User priorities									
	Likert scale					Ranking task				
	Kinematics	Spatio-temporal	Balance	User perception	Muscular control	Kinematics	Spatio-temporal	Balance	User perception	Muscular control
CP01	4	5	5	4	4		1	2		3
CP02	5	3	5	4	4	2		1		3
CP03	5	5	5	5	5			1	3	2
CP04	4	4	5	5	5		1	2	3	
CP05	3	4	5	5	5			3	2	1
CP06	5	3	4	4	5	1		2		3
CP07	5	4	4	5	5			2	1	3
CP08	5	4	4	5	4	1	2	3		
CP09	3	5	4	2	3	1	2		3	
CP10	4	5	5	5	5			2	1	3

Participant ID	Physiotherapist priorities									
	Likert scale					Ranking task				
	Kinematics	Spatio-temporal	Balance	User perception	Muscular control	Kinematics	Spatio-temporal	Balance	User perception	Muscular control
CP01	5	4	4	4	4	1	2		3	
CP02	5	5	3	4	4	1	2			3
CP03	5	5	5	5	5	1		3	2	
CP04	4	3	4	5	5	3			2	1
CP05	5	4	3	5	4	2	1		3	
CP06	5	4	5	5	4	1		3	2	
CP07	5	5	5	5	4	1		2	3	
CP08	4	5	4	5	5	3		1		
CP09	3	3	4	2	4	2	3	1		
CP10	5	3	4	4	5			3	2	1

Empty cells indicate unranked items

## Appendix B

The influence of the $\alpha$ term (Eq. 10) is shown in Fig. 6, where the total error and the final optimal stiffness are computed for $\alpha =0.5$ and $\alpha =0$. These results can be compared with the ones in Fig. 5 ($\alpha =1$).

## Abbreviations

AFO: Ankle-foot orthosis
CP: Cerebral palsy
DF: Dorsiflexion
PF: Plantarflexion
HILO: Human in the loop optimization
VSO: Variable stiffness orthosis
STA: Step time asymmetry
PCI: Physiological cost index
CCI: Co-contraction index
TA: Tibialis anterior
SOL: Soleus
GL: Gastrocnemius lateralis
GM: Gastrocnemium mediales
TD: Typical development
RMSE: Root mean squared error
GMFCS: Gross Motor Function Classification System
HNJ: Hospital Niño Jesús
MWT: Two minutes walking test
FSR: Force sensitive resistors
IMU: Inertial measurement unit
EMG: Electromyography
